# Supplementary material for: Costimulation of type-2 innate lymphoid cells by GITR promotes effector function and ameliorates type 2 diabetes
Source: Nat Commun. 2019 Feb 12;10:713. doi: 10.1038/s41467-019-08449-x (PMC6372786; doi:10.1038/s41467-019-08449-x)
Supplement: Supplementary file 1 — Supplementary Figures [file 41467_2019_8449_MOESM1_ESM.pdf]

Costimulation of ILC2s by GITR promotes effector function and ameliorates type 2 diabetes

Galle-Treger, et al.

Supplementary Figure 1

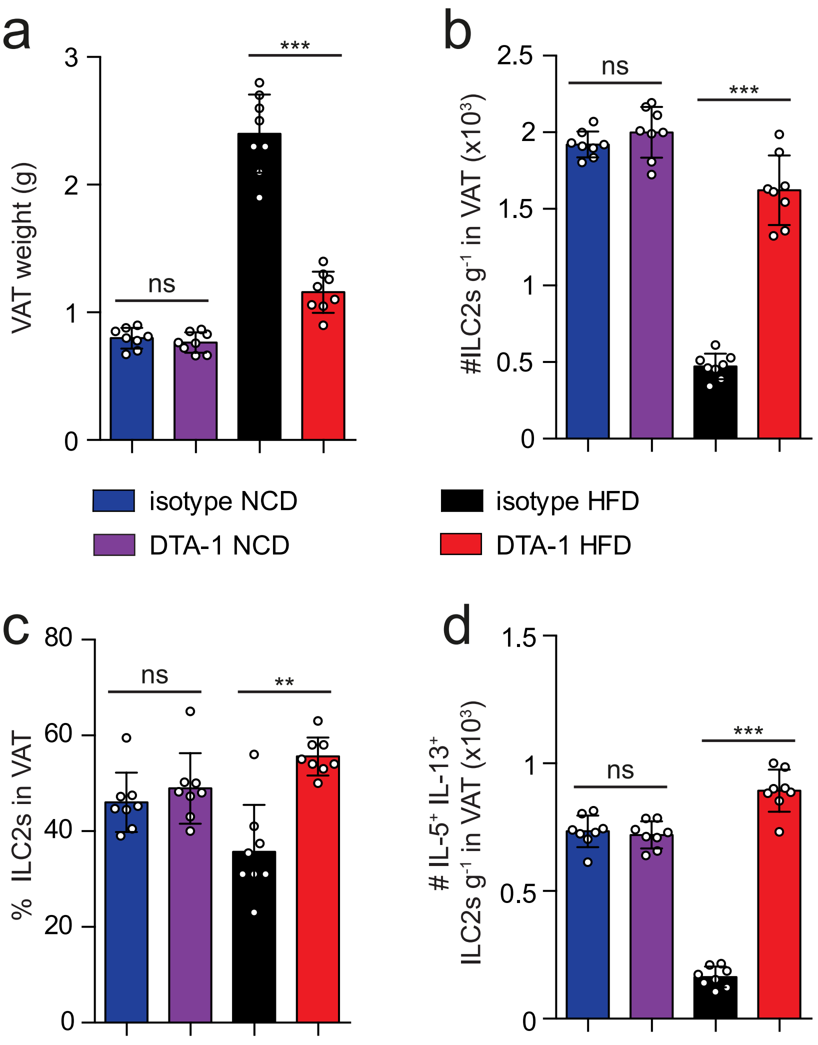

Engagement of GITR protects from the onset of Type 2 Diabetes. A cohort of C57/BL6 mice fed a normal chow diet (NCD) or a high fat diet (HFD) were either treated with DTA-1 (1mg/mouse) or isotype control by intraperitoneal injections every four days, n=8. VAT weight (a) after 14 weeks of treatment. The number (b) and frequency (c) of VAT resident ILC2s after 14 weeks of treatment. Number of IL-5^{+} IL-13^{+} ILC2s per gram of VAT after 14 weeks of treatment. Error bars are the mean ± SEM. Student's t-test, **$p<0.01$, ***$p<0.001$, ns: non-significant.

Supplementary Figure 2

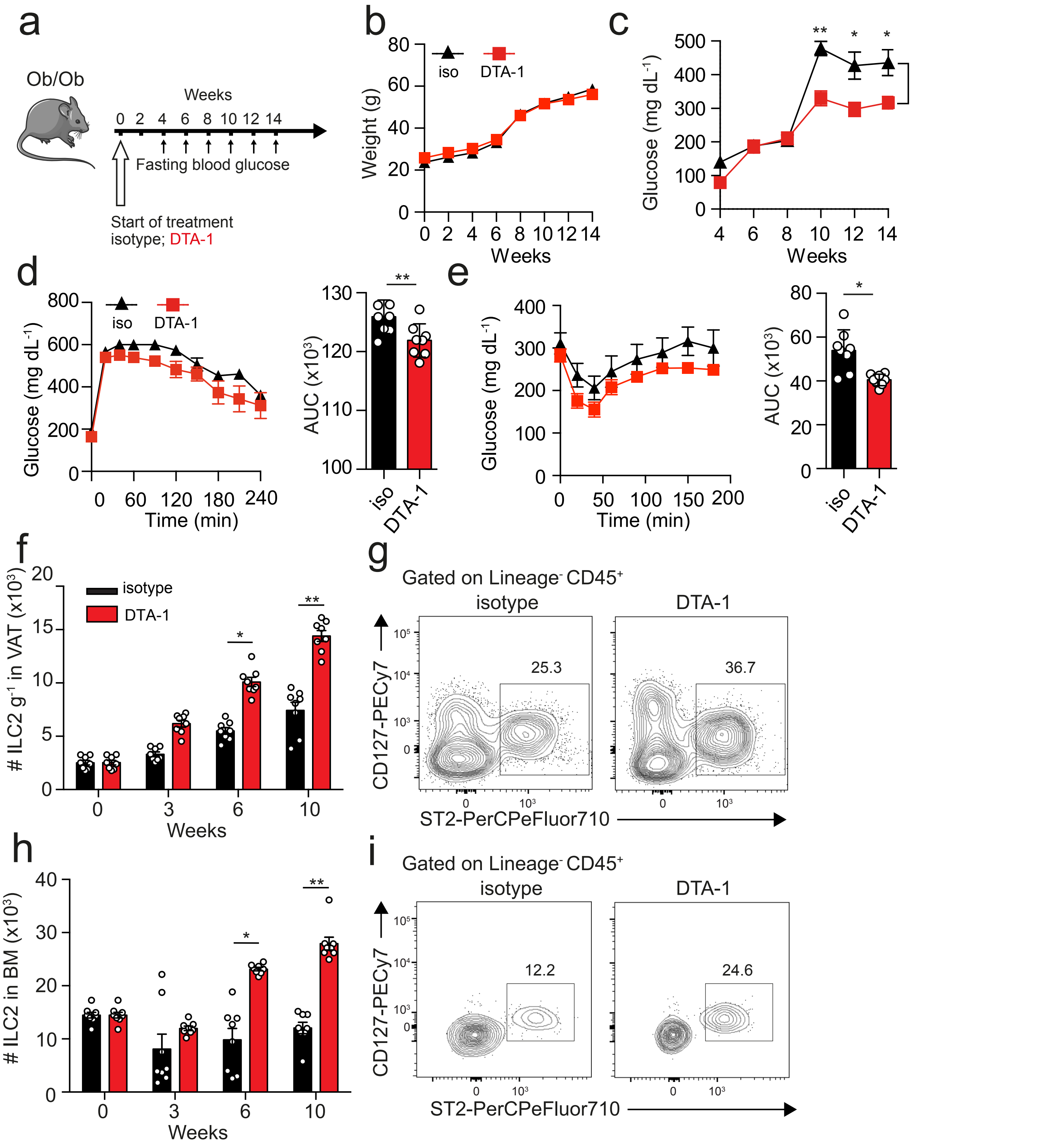

Engagement of GITR protects from the onset of Type 2 Diabetes. (a) A cohort of Ob/Ob mice were either treated with DTA-1 (1mg/mouse) or isotype control by intraperitoneal injections every four days according to the scheme, n=8. (b) Total weight and (c) fasting blood glucose levels were measured every two weeks for 14 weeks. Glucose tolerance test (d) and insulin tolerance test (e) were performed in a cohort of Ob/Ob mice after 14 weeks of treatment. The area under the curve was calculated for each group. Mice were euthanized on 0, 3, 6 and 10 weeks and VAT (f) and BM (h) resident ILC2s were quantified at the indicated times, n=8 (f). Representative FACS plots of VAT (g) and BM (i) $Lin^{-}CD45^{+}$ $IL\text{-}7R^{+}ST2^{+}$ ILC2s on week 10 are shown. Error bars are the mean ± SEM. Student's t-test, *p<0.05, **p<0.01, ***p<0.001. Mouse image provided with permission from Servier Medical Art.

Supplementary Figure 3

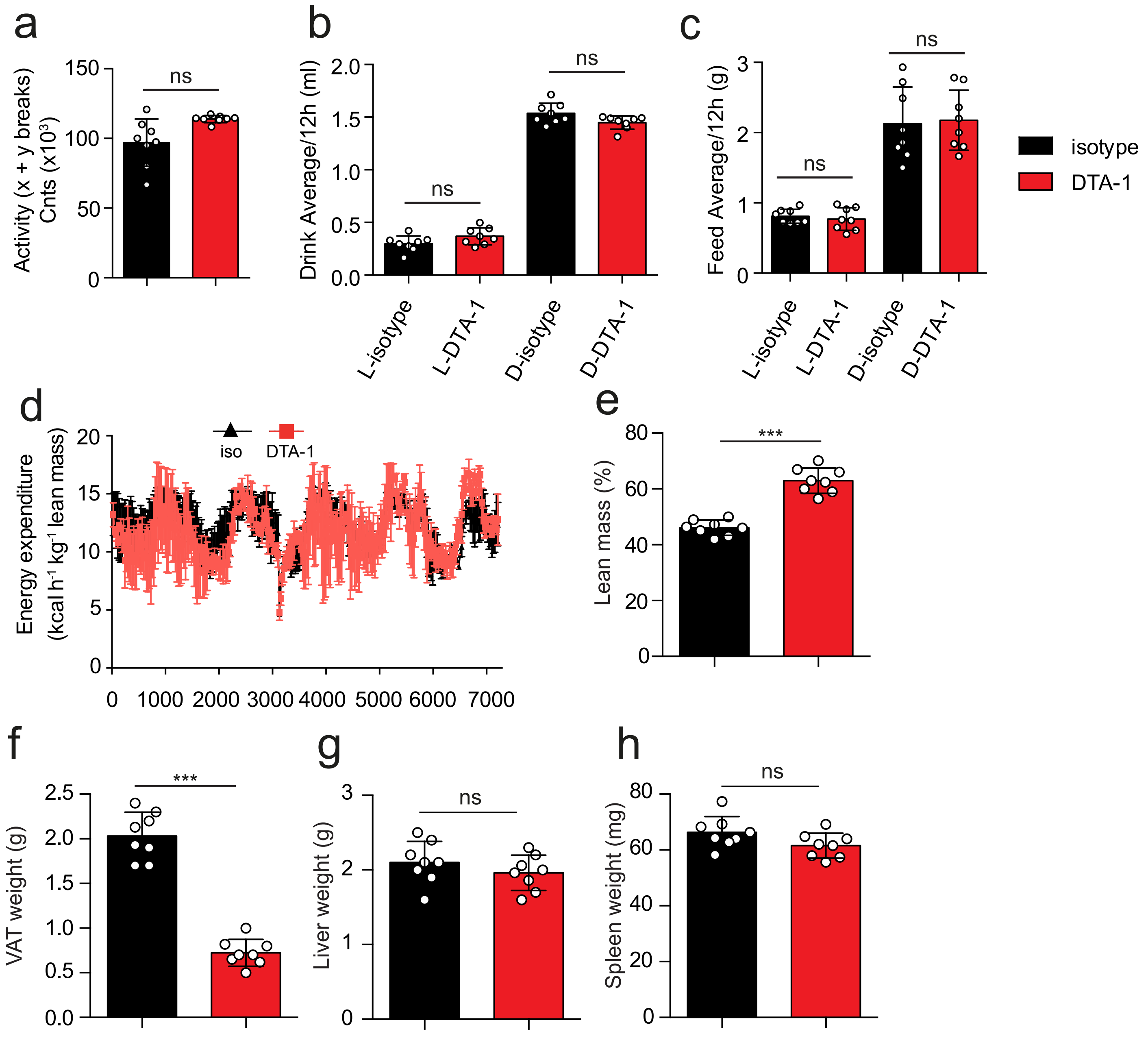

Effects of GITR engagement on physical activity, water, food intake, VAT weight and lean mass percentage in Rag2$^{-/-}$ mice. A cohort of Rag2$^{-/-}$ mice were fed on HFD and either treated with DTA-1 (1mg/mouse) or isotype control by intraperitoneal injection every four days. CLAMS analysis was performed using individually housed groups of DTA-1 or isotype control treated Rag2$^{-/-}$ mice maintained on a HFD. After 14 weeks of HFD, we measured physical activity (a), water (b) or food (c) intake and energy expenditure normalized by the lean mass (d). D: Dark, L: Light. The percentage of lean mass (e) was measured by using a body composition analyzer. After 14 weeks of treatment, mice were euthanized and the VAT (f), liver (g) and spleen (h) were respectively isolated and weighed. Data are expressed as means +/- SEM, n=8. Student's t-test, ***p<0.001, ns:non-significant.

Supplementary Figure 4

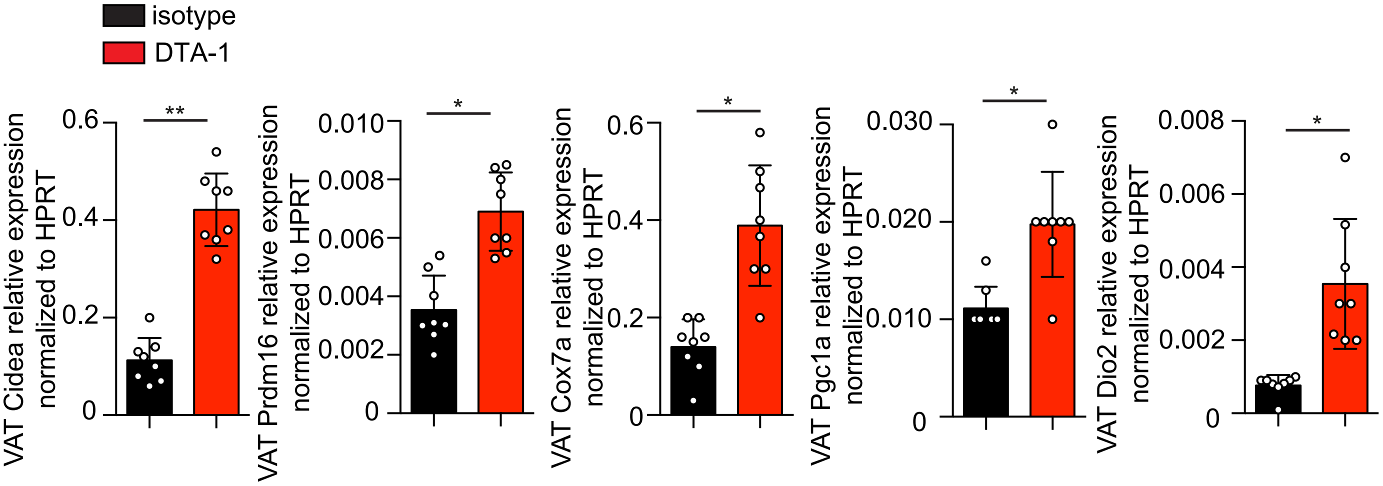

Effects of GITR engagement on the expression of VAT browning-associated genes. A cohort of Rag2$^{-/-}$ mice fed HFD were either treated with DTA-1 (1mg/mouse) or isotype control by intraperitoneal injection every four days and euthanized after 14 weeks of treatment. mRNA expression of VAT lysates was determined by qRT-PCR using specific primers for Cidea, Prdm16, Pgc1a, Cox7a, Dio2 and hypoxanthine-guanine phosphoribosyltransferase (Hprt). Data are expressed as means +/- SEM, n=8. Student's t-test, $*p<0.05$.

Supplementary Figure 5

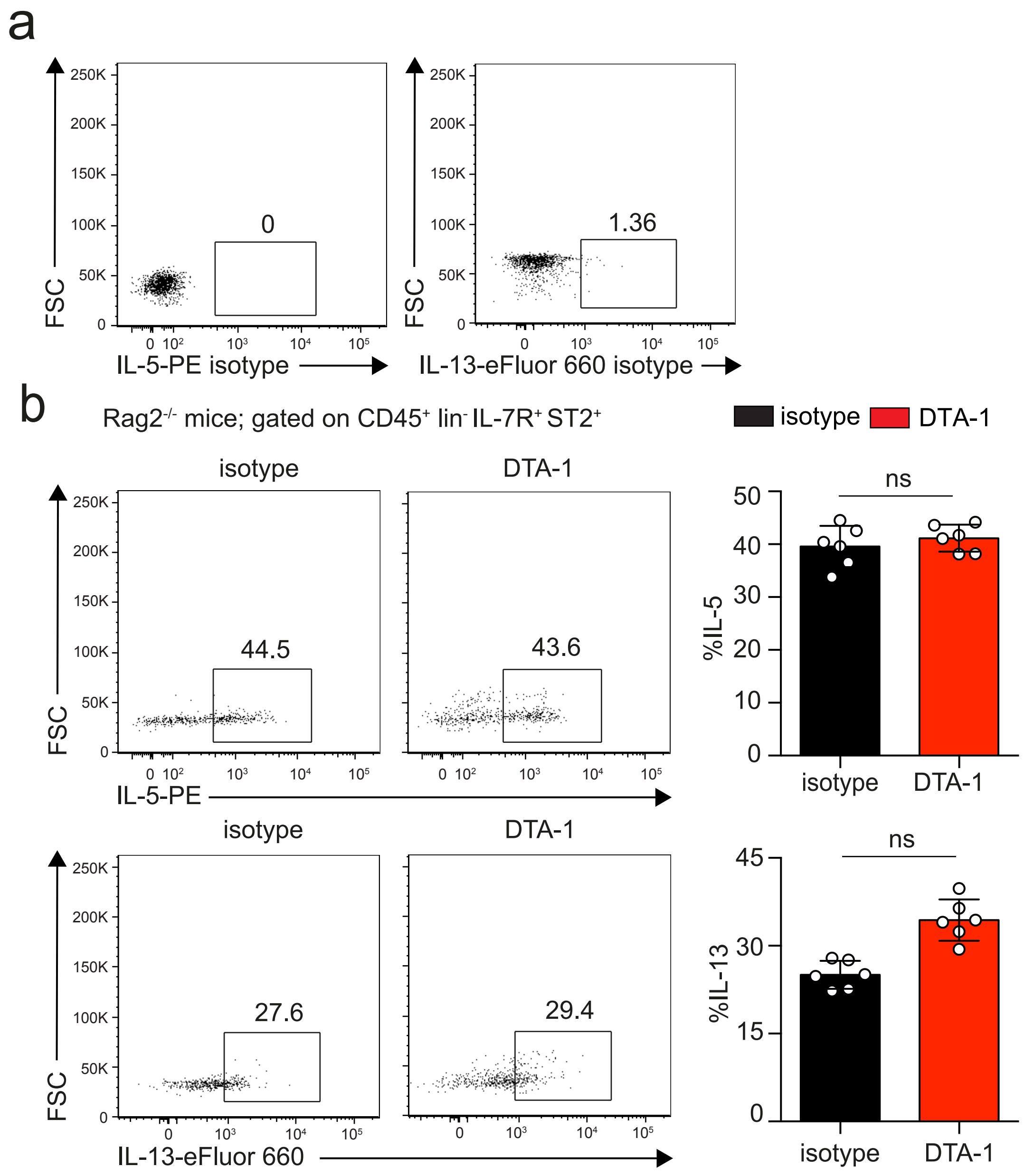

GITR engagement does not induce Th2-cytokines in ILC2s in lean Rag2$^{-/-}$ mice.
(a) Representative flow cytometry plots of ILC2 intracellular staining with IL-5 (left) and IL-13 (right) isotype control antibodies. (b) A cohort of Rag2$^{-/-}$mice were fed on chow diet and either treated with isotype control or DTA-1 (1mg/mouse) by intraperitoneal injection for 6 consecutive days. Representative flow cytometry plots of intracellular IL-5 (top panel) and IL-13 (bottom panel) in VAT ILC2s and corresponding quantitation, presented as frequency of positive IL-5 and IL-13 ILC2s respectively, n=6. Error bars are the mean ± SEM. Student's t-test, ns: non-significant.

Supplementary Figure 6

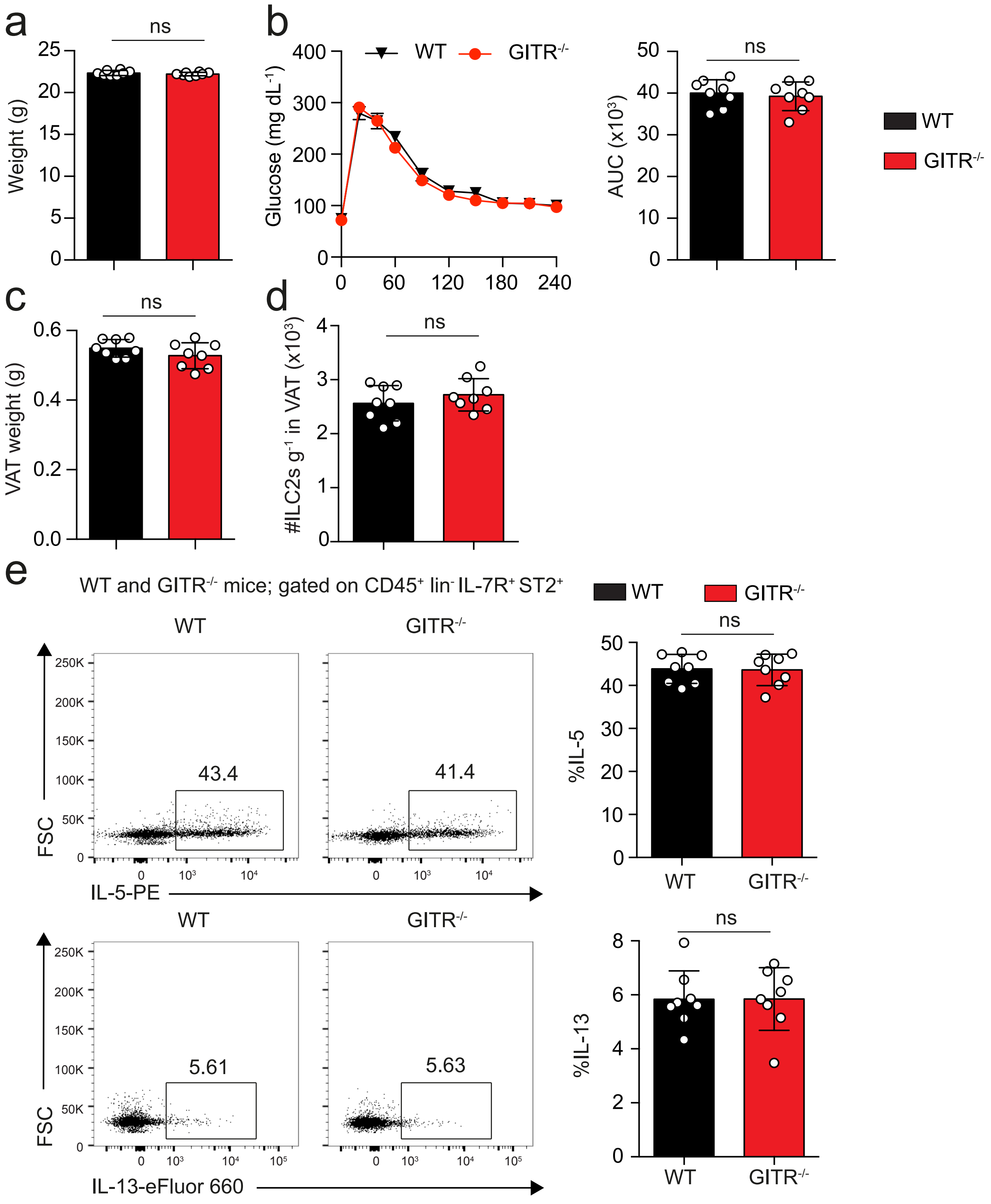

Deletion of GITR has no effect on glucose and ILC2 homeostasis in lean mice. A cohort of WT and GITR$^{-/-}$ mice were fed a NCD for 10 weeks, n=8. Total weight was measured after 10 weeks on a NCD (a). Glucose tolerance test (b) was performed in a cohort of WT and GITR$^{-/-}$ mice at 10 weeks old. The area under the curve was calculated for each group. Mice were euthanized at 10 weeks old and VAT weight (c) was measured and VAT resident ILC2s (d) were quantified, n=8. (e) Representative flow cytometry plots of intracellular IL-5 (top panel) and IL-13 (bottom panel) in VAT ILC2s and corresponding quantitation, presented as frequency of positive IL-5 and IL-13 ILC2s respectively, n=8, Error bars are the mean ± SEM. Student's t-test, ns: non-significant.

Supplementary Figure 7

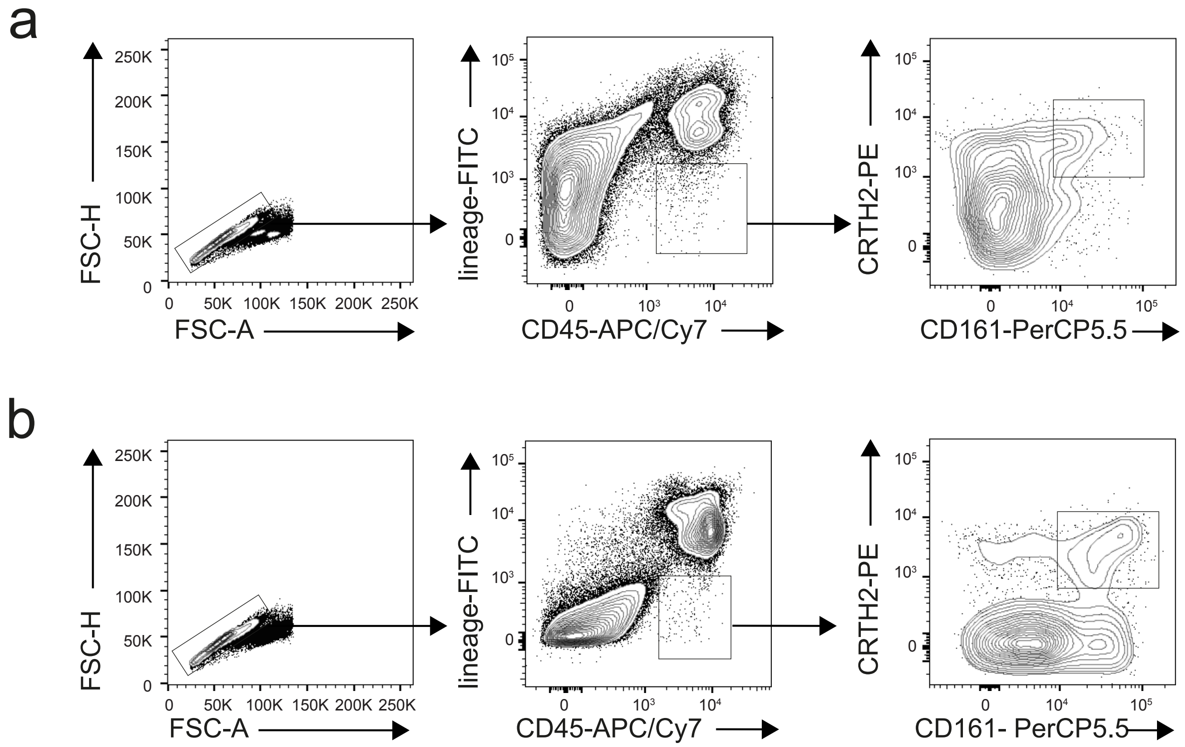

ILC2 gating strategies in human blood and adipose tissue. Gating strategy of Lin$^-$CD45$^+$CRTH2$^+$CD161$^+$ ILC2 cells in blood (a) and in adipose tissue (b).
